# C-2 phenyl replacements to obtain potent quinoline-based *Staphylococcus aureus* NorA inhibitors

**DOI:** 10.1080/14756366.2020.1719083

**Published:** 2020-01-29

**Authors:** Tommaso Felicetti, Gianmarco Mangiaterra, Rolando Cannalire, Nicholas Cedraro, Donatella Pietrella, Andrea Astolfi, Serena Massari, Oriana Tabarrini, Giuseppe Manfroni, Maria Letizia Barreca, Violetta Cecchetti, Francesca Biavasco, Stefano Sabatini

**Affiliations:** a Department of Pharmaceutical Sciences, Chemistry and Technology of the Drug Section, Università degli Studi di Perugia, Perugia, Italy; b Department of Life and Environmental Sciences, Università Politecnica delle Marche, Ancona, Italy; c Department of Pharmaceutical Sciences, Biochemical Sciences and Health Section, Università degli Studi di Perugia, Perugia, Italy

**Keywords:** Antimicrobial resistance breakers, efflux pump inhibitors, NorA, *Staphylococcus aureus*, antimicrobial resistance

## Abstract

NorA is the most studied efflux pump of *Staphylococcus aureus* and is responsible for high level resistance towards fluoroquinolone drugs. Although along the years many NorA efflux pump inhibitors (EPIs) have been reported, poor information is available about structure-activity relationship (SAR) around their nuclei and reliability of data supported by robust assays proving NorA inhibition. In this regard, we focussed efforts on the 2-phenylquinoline as a promising chemotype to develop potent NorA EPIs. Herein, we report SAR studies about the introduction of different aryl moieties on the quinoline C-2 position. The new derivative **37a** showed an improved EPI activity (16-fold) with respect to the starting hit **1**. Moreover, compound **37a** exhibited a high potential in time-kill curves when combined with ciprofloxacin against SA-1199B (*norA+)*. Also, **37a** exhibited poor non-specific effect on bacterial membrane polarisation and showed an improvement in terms of “selectivity index” in comparison to **1**.

## Introduction

Antimicrobial resistance (AMR) is a complex threat for human health and represents a hot topic in drug discovery^1^. The use of large amounts of antibiotics to control human and animal infections and in animal breeding has created unprecedented conditions for the rising and spread of antibiotic resistance among bacterial populations. The “right drug for the right bug” approach remains a distant perspective, currently replaced by an empirically-guided consumption. Recently, the World Health Organisation (WHO) has included AMR in the “ten threats to global health in 2019”, forecasting an imminent return to a time when we were unable to easily treat common infections^2^. Considering the microbial promptness in achieving successful machinery escaping antibiotic activity also towards new drugs^3^^,^^4^, the use of non-antibiotic adjuvant molecules targeting resistance mechanisms, in co-administration with antibacterials, is a valid approach to recover drug sensitivity in resistant strains^5^^,^^6^. The fascinating idea to “freeze” resistance would allow antibiotics, for which resistance occurred, to recover their activity thereby renewing our armamentarium to fight microbial infections. Amongst the wide range of resistance mechanisms developed by bacteria, one of the most common is the drug extrusion from the cell, which can reduce intracellular drugs to sub-inhibitory concentrations allowing bacteria to grow in the presence of routinely adopted therapeutic doses^7^. Indeed, for some drugs, microorganisms can only acquire resistance in the presence of efflux pump activity. Most likely, efflux pumps play a non-specific role in the early stages of antibiotic exposure, thereby allowing microorganisms to develop more specific and effective mechanisms of resistance^4^^,^^8^^,^^9^. Therefore, the use of efflux pump inhibitors (EPIs) in combination with extruded drugs may be a major strategy in the development of effective antimicrobial treatments. To date, little has been done in terms of EPI development and no inhibitors have ever reached the clinical use^10^.

Among the six multi-drug resistant bacterial species termed ESKAPE pathogens (*Enterococcus faecium*, *Staphylococcus aureus*, *Klebsiella pneumoniae*, *Acinetobacter baumannii*, *Pseudomonas aeruginosa* and *Enterobacter species*), *S. aureus* and its methicillin-resistant strain (MRSA) represent a serious problem worldwide, due to its acquired resistance to several classes of antibiotics, encoded by the SCC-*mec* cassettes^11^. Moreover, the trans-membrane protein NorA, belonging to the Major Facilitator Superfamily, is commonly overexpressed in *S. aureus* resistant strains and strongly upregulated in response to fluoroquinolones treatment. NorA can extrude different toxic compounds, including the fluoroquinolone ciprofloxacin (CPX) and the dye ethidium bromide (EtBr), by an antiporter mechanism exploiting the proton motive force^12^.

Along the years many NorA EPIs have been discovered by three different approaches: i) screening libraries of natural or synthetic molecules; ii) repurposing molecules with known biological activity and iii) designing and synthesising new compounds^10^^,^^13–16^. The lack of NorA structural information has strongly hampered the identification of potent NorA EPIs. No examples of structure-based drug design have been so far reported for EPI identification, which, therefore, relies on ligand-based drug design approaches or classical medicinal chemistry strategies. In addition, the risk for a strategy aimed at identifying an EPI relies on the poor availability of quick and smart biological screenings able to early identify active molecules. On the contrary, due to the poor knowledge of the NorA efflux mechanisms and the lack of biophysical experiments validating a true NorA inhibition, too frequently molecules have been described in literature as NorA EPIs when they are not. The ability of a compound to act in synergy with CPX against *S. aureus* resistant strains seems not to be sufficient to consider that molecule as NorA EPI. Additional experiments are needed to rule out non-specific effects of compounds boosting antibiotic activity. Since many efflux pumps work through the proton motive force, its disruption by depolarising the bacterial membrane is the most common example of a non-specific effect still resulting in efflux pump inhibition. However, selective membrane depolarisation in microorganisms appears challenging and often compounds result very toxic by eliciting the same effect on eukaryotic cells. Conversely, an actual NorA EPI must exert a strong synergistic effect with CPX (reducing its MIC at least 4-fold) against overexpressing *norA S. aureus* strains while resulting poorly active or ineffective against both wild-type and *norA* knock-out strains. In addition, NorA efflux inhibition activity should be demonstrated by phenotypic assays (i.e. EtBr efflux assays) in *norA* overexpressing *S. aureus* strains and non-specific EPI effect needs to be excluded by performing bacterial membrane polarisation experiments. Moreover, the identified EPIs should not possess any intrinsic antibacterial effect at the concentrations needed to reach synergism with antibiotics in order to prevent a potential interference throughout the synergism with antibacterials.

**Figure 1.**
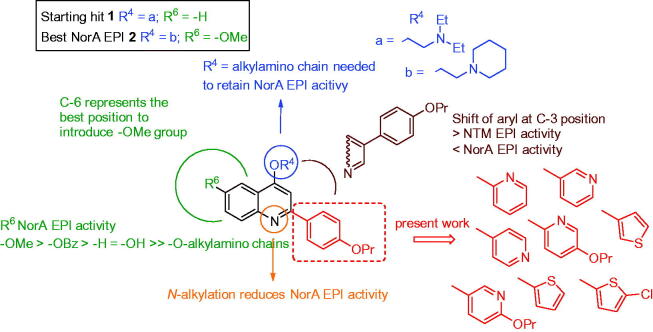
Known SAR around the 2-phenylquinoline scaffold and new designed compounds.

In this direction, we are currently working on 2-phenylquinoline nucleus giving rise to a really promising class of NorA EPIs. 2-Phenylquinoline derivatives have shown an excellent NorA EPI activity, widely restoring CPX antibacterial activity against resistant *S. aureus* strains. Efforts aimed at delineating a robust structure-activity relationship (SAR) investigation around this scaffold highlighted some significant findings: i) an alkylamino chain on the oxygen at quinoline C-4 position is needed to retain NorA EPI activity and preferred over *N*-1 position^17^^,^^18^; ii) the introduction of a methoxy group on the C-6 position of the 2-phenylquinoline core strongly improved NorA EPI activity while lowering host cell toxicity^19^; iii) the replacement of the methoxy group at C-6 position with a benzyloxy moiety retained NorA inhibition activity (although increasing human cell toxicity), while a free hydroxy group or different *O*-alkylamino chains at C-6 yielded less potent analogues^20^; iv) the shift of the aryl portion from C-2 to C-3 position of the quinoline core increased EPI activity towards nontuberculous mycobacteria (NTM) while decreasing that against *S. aureus* (Figure 1)^21^^,^^22^.

In this work, we focus efforts on the exploration of the C-2 position by replacing the 4’-proproxyphenyl substituent with differently substituted pyridine or thiophene moieties in order to identify potential isosteric replacements (Figure 1). Indeed, previous SAR studies did not cover such a portion on the quinoline scaffold. Although more potent 2-phenylquinoline-based NorA EPIs have been identified by us, we selected the previously reported compound **1** as starting hit^23^, because the lack of substituents on the quinoline benzene ring allows for a better comparison with the new C-2 modified analogues (Figure 1). Considering the role of *O*-alkylamino chains on the quinoline C-4 position to retain NorA EPI activity, we introduced two different chains (a, *O*-ethyl-*N*,*N*-diethylamino and b, *O*-ethylpiperidine) for each new C-2 aryl quinoline scaffold. In particular, chain “a” was selected because present in the starting hit **1** and chain “b” since it resulted the best chain in the last work, where the most potent methoxy 2-phenylquinoline derivative **2** was identified (Figure 1)^19^.

## Materials and methods

### Chemistry

All starting materials, reagents and solvents were purchased from common commercial suppliers and were used as such, unless otherwise indicated. Organic solutions were dried over anhydrous Na_2_SO_4_ and concentrated with a rotary evaporator at low pressure. All reactions were routinely checked by thin-layer chromatography (TLC) on silica gel 60F_254_ (Merck) and visualised by using UV or iodine. Flash chromatography separations were carried out on Merck silica gel 60 (mesh 230–400). Melting points were determined in capillary tubes (Stuart SMP30) and are uncorrected. Yields were of purified products and were not optimised. ^1^H NMR spectra were recorded at 200 or 400 MHz (Bruker Avance DRX-200 or 400, respectively) while ^13^C NMR spectra were recorded at 101 MHz (Bruker Avance DRX-400). Chemical shifts are given in ppm (*δ*) relative to TMS. Spectra were acquired at 298 K. Data processing was performed with standard Bruker software XwinNMR and the spectral data are consistent with the assigned structures. The purity of the tested compounds was evaluated by combustion analysis using a Fisons elemental analyser, model EA1108CHN, and data for C, H, and N are within 0.4% of the theoretical values (≥95% sample purity).

#### *N*-(2-acetylphenyl)-5-propoxypyridine-2-carboxamide (14)

SOCl_2_ (1.2 mL, 16.44 mmol) was slowly added at 0 °C to 5-propoxypyridine-2-carboxylic acid **3** (0.39 g, 2.20 mmol), then the reaction mixture was stirred at 60 °C for 30 min. The excess of SOCl_2_ was removed under reduced pressure to give 5-propoxypyridine-2-carbonyl chloride (**5**) as a yellow oil that immediately was dissolved in dry THF (6 mL) and added to a mixture of aminoacetophenone **13** (0.29 g, 2.20 mmol) and Et_3_N (0.9 mL, 6.60 mmol) in dry THF (14 mL). The reaction was stirred at rt for 2 h, then it was poured in ice/water and the pH was adjusted to ≃8 with 2 N HCl. The mixture was extracted by EtOAc and the organic layers were washed by brine, dried over Na_2_SO_4_ and evaporated to dryness. After purification by flash chromatography column (CH_2_Cl_2_/acetone 98/2), derivative **14** was obtained as a yellow solid (0.41 g, 63% yield), m.p. 132.0–134.0 °C. ^1^H NMR (200 MHz, DMSO-d_6_): *δ* = 1.02 (t, *J* = 7.5 Hz, 3H, OCH_2_CH_2_*CH_3_*), 1.73–1.87 (m, 2H, OCH_2_*CH_2_*CH_3_), 2.59 (s, 3H, CH_3_), 4.00 (t, *J* = 6.6 Hz, 2H, O*CH_2_*CH_2_CH_3_), 7.11 (t, *J* = 7.6 Hz, 1H, H-4’), 7.23–7.28 (m, 1H, H-4), 7.56 (t, *J* = 8.4 Hz, 1H, H-5’), 7.90 (d, *J* = 7.9 Hz, 1H, H-6’), 8.15 (d, *J* = 8.5 Hz, 1H, H-3’), 8.39 (d, *J* = 2.1 Hz, 1H, H-6), 8.96 (d, *J* = 8.7 Hz, 1H, H-3), 13.37 ppm (s, 1H, NH).

#### *N*-(2-acetylphenyl)-6-propoxynicotinamide (15)

SOCl_2_ (0.6 mL, 8.22 mmol) was slowly added at 0 °C to 6-propoxynicotinic acid **4** (0.20 g, 1.10 mmol), then the reaction mixture was stirred at 60 °C for 30 min. The excess of SOCl_2_ was removed under reduced pressure to give 6-propoxynicotinoyl chloride (**6**) as a yellow oil that immediately was dissolved in dry THF (4 mL) and added to a mixture of aminoacetophenone **13** (0.15 g, 1.10 mmol) and Et_3_N (0.7 mL, 5.50 mmol) in dry THF (10 mL). The reaction was stirred at rt for 2 h, then it was poured in ice/water and the pH was adjusted to ≃8 with 2 N HCl. The mixture was extracted by EtOAc and the organic layers were washed by brine, dried over Na_2_SO_4_ and evaporated to dryness. After purification by flash chromatography column (CH_2_Cl_2_/acetone 98/2), derivative **15** was obtained as a brown oil (0.18 g, 56% yield). ^1^H NMR (200 MHz, DMSO-d_6_): *δ* = 0.81 (t, *J* = 7.3 Hz, 3H, OCH_2_CH_2_*CH_3_*), 1.57–1.72 (m, 2H, OCH_2_*CH_2_*CH_3_), 2.60 (s, 3H, CH_3_), 3.89 (t, *J* = 7.3 Hz, 2H, O*CH_2_*CH_2_CH_3_), 6.48 (d, *J* = 8.7 Hz, 1H, H-6’), 7.18 (t, *J* = 7.5 Hz, 1H, H-4’), 7.59 (t, *J* = 7.7 Hz, 1H, H-5’), 7.78–7.84 (m, 1H, H-5), 8.00 (d, *J* = 8.0 Hz, 1H, H-3’), 8.38–8.41 (m, 2H, H-2 and H-4), 11.91 ppm (s, 1H, NH).

#### General procedure A for the synthesis of compounds 16–21

A solution of acyl chlorides **7–12** (1 equiv.) in dry THF (4 ml per mmol) was added to a solution of aminoacetophenone **13** (1 equiv) and Et_3_N (5 equiv.) in dry THF (5 ml per mmol of **13**). The reaction was stirred at rt for 90 min–72 h, then it was poured in ice/water. The pH was adjusted up to ≃8 with 2 N HCl, the mixture was extracted by EtOAc and the organic layer was washed by brine, dried over Na_2_SO_4_ and evaporated under vacuum to a solid that was purified by flash column chromatography.

#### *N*-(2-acetylphenyl)pyridine-2-carboxamide (16)

General procedure A: time, 90 min; used chloride, pyridine-2-carbonyl chloride **7** (0.57 g, 4.06 mmol); purification, (EP/EtOAc 60/40). Derivative **16**^24^ was obtained as a grey solid (0.71 g, 73% yield), mp 107.5–108.0 °C. ^1^H NMR (400 MHz, CDCl_3_): *δ* = 2.69 (s, 3H, CH_3_), 7.27 (t, *J* = 7.3 Hz, 1H, H-4’), 7.67–7.71 (m, 2H, H-5’ and H-6’), 8.06–8.20 (m, 3H, H-3, H-5 and H-3’), 8.76–8.87 (m, 2H, H-4 and H-6), 13.31 ppm (s, 1H, NH).

#### *N*-(2-acetylphenyl)nicotinamide (17)

General procedure A: time, 72 h; used chloride, nicotinoyl chloride **8** (0.57 g, 4.06 mmol); purification, (EP/EtOAc 60/40). Derivative **17**^24^ was obtained as a white solid (0.96 g, 99% yield), mp 114.0–115.0 °C. ^1^H NMR (200 MHz, CDCl_3_): *δ* = 2.69 (s, 3H, CH_3_), 7.12 (m, 1H, H-4’), 7.37–7.42 (m, 1H, H-4), 7.61 (dt, *J* = 1.5 and 8.7 Hz, 1H, H-5’), 7.95 (dd, *J* = 1.4 and 8.3 Hz, 1H, H-3’), 8.27–8.33 (m, 1H, H-5), 8.75 (dd, *J* = 1.8 and 8.5 Hz, 1H, H-6’), 8.90 (d, *J* = 8.4 Hz, 1H, H-6), 9.27 (d, *J* = 1.9 Hz, 1H, H-2), 12.73 ppm (s, 1H, NH).

#### *N*-(2-acetylphenyl)isonicotinamide (18)

General procedure A: time, 20 h; used chloride, isonicotinoyl chloride **9** (3.45 g, 24.40 mmol); purification, (EP/EtOAc 50/50). Derivative **18**^24^ was obtained as a white solid (3.52 g, 60% yield), mp 111.0–112.0 °C. ^1^H NMR (200 MHz, CDCl_3_): *δ* = 2.66 (s, 3H, CH_3_), 7.16 (dt, *J* = 1.2 and 7.9 Hz, 1H, H-4’), 7.61 (dt, *J* = 1.6 and 7.4 Hz, 1H, H-5’), 7.83–7.86 (m, 2H, H-3 and H-5), 7.94 (dd, *J* = 1.5 and 8.0 Hz, 1H, H-6’), 8.73–8.80 (m, 2H, H-2 and H-6), 8.89 (dd,*J* = 1.1 and 8.5 Hz, 1H, H-3’), 12.85 ppm (s, 1H, NH).

#### *N*-(2-acetylphenyl)thiophene-2-carboxamide (19)

General procedure A: time, 20 h; used chloride, thiophene-2-carbonyl chloride **10** (0.34 g, 2.34 mmol); purification, (EP/EtOAc 50/50). Derivative **19**^24^ was obtained as a yellow solid (0.33 g, 58% yield), mp129.0–131.0 °C. ^1^H NMR (200 MHz, CDCl_3_): *δ* = 2.68 (s, 3H, CH_3_), 7.04–7.15 (m, 2H, H-4 and H-4’), 7.51–7.61 (m, 2H, H-3 and H-5’), 7.79 (dd, *J* = 1.1 and 5.0 Hz, 1H, H-5), 7.91 (dd, *J* = 1.6 and 8.0, 1H, H-6’), 8.83 (dd, *J* = 1.2 and 8.5 Hz, 1H, H-3’), 12.70 ppm (s, 1H, NH).

#### *N*-(2-acetylphenyl)thiophene-3-carboxamide (20)

General procedure A: time, 12 h; used chloride, thiophene-3-carbonyl chloride **11** (0.41 g, 2.80 mmol); purification (EP/EtOAc 50/50). Derivative **20**^24^ was obtained as a white solid (0.34 g, 50% yield), mp 83.0–84.0 °C. ^1^H NMR (200 MHz, CDCl_3_): *δ* = 2.67 (s, 3H, CH_3_), 7.10 (dt, *J* = 1.1 and 7.2 Hz, 1H, H-4’), 7.35 (dd, *J* = 3.0 and 5.5 Hz, 1H, H-4), 7.57 (dt, *J* = 1.6 and 8.6 Hz, 1H, H-5’), 7.63 (dd, *J* = 1.1 and 5.3 Hz, 1H, H-2), 7.91 (dd, *J* = 1.6 and 8.0 Hz, 1H, H-6’), 8.11 (dd, *J* = 1.1 and 3.0 Hz, 1H, H-5), 8.88 (dd, *J* = 1.1 and 8.4 Hz, 1H, H-3’), 12.57 (s, 1H, NH).

#### *N*-(2-acetylphenyl)-5-chlorothiophene-2-carboxamide (21)

General procedure A: time, 12 h; used chloride, 5-chlorothiophene-2-carbonyl chloride **12** (0.56 g, 3.08 mmol); purification, (EP/EtOAc 50/50). Derivative **21** was obtained as a white solid (0.73 g, 85% yield), mp 136.0–137.0 °C. ^1^H NMR (200 MHz, CDCl_3_): *δ* = 2.67 (s, 1H, CH_3_), 6.93 (d, *J* = 6.1 Hz, 1H, H-3), 7.11 (t, *J* = 7.6 Hz, 1H, H-4’), 7.52–7.62 (m, 2H, H-4 and H-5’), 7.91 (d, *J* = 8.0 Hz, 1H, H-6’), 8.77 (d, *J* = 8.4 Hz, H-3’), 12.66 ppm (s, 1H, NH).

#### General procedure B for the synthesis of compounds 22–29

Under N_2_ atmosphere in a pressure tube, NaOH powder (3 equiv) was added to a solution of derivatives **14–21** (1 equiv) in dry dioxane (2 ml per mmol). The reaction mixture was stirred at 110 °C for 2–8 h, then it was poured in ice/water. The pH was acidified up to 6 with 2 N HCl and the obtained precipitate was filtered to give the desired compounds as solids. After crystallization by Et_2_O/EtOH, compounds were used for the next reactions.

#### 2–(5-Propoxypyridin-2-yl)quinolin-4-ol (22)

General procedure B: time, 6 h; starting material, *N*-(2-acetylphenyl)-5-propoxypyridine-2-carboxamide **14** (0.23 g, 0.73 mmol). Compound **22** was obtained as a white solid (0.20 g, 100% yield), mp 190.0–191.0 °C. ^1^H NMR (400 MHz, DMSO-d_6_): *δ* = 1.95 (t, *J* = 7.4 Hz, 3H, OCH_2_CH_2_*CH_3_*), 3.42–3.60 (m, 2H, OCH_2_*CH_2_*CH_3_), 4.09 (t, *J* = 6.6 Hz, 2H, O*CH_2_*CH_2_CH_3_), 6.80 (s, 1H, H-3), 7.29 (t, *J* = 7.4 Hz, 1H, H-6), 7.56–7.64 (m, 2H, H-7 and H4’), 8.00 (d, *J* = 8.4 Hz, 1H, H-3’), 8.05 (d, *J* = 7.4 Hz, 1H, H-8), 8.20 (d, *J* = 8.8 Hz, 1H, H-5), 8.45 (d, *J* = 2.7 Hz, 1H, H-6’), 11.81 ppm (s, 1H, OH).

#### 2–(6-Propoxypyridin-3-yl)quinolin-4-ol (23)

General procedure B: time, 6 h; starting material, *N*-(2-acetylphenyl)-6-propoxynicotinamide **15** (0.75 g, 2.51 mmol). Compound **23** was obtained as a white solid (0.40 g, 57% yield), mp 212.5–215.0 °C.^1^H NMR (400 MHz, DMSO-d_6_): *δ* = 0.86 (t, *J* = 7.5 Hz, 3H, OCH_2_CH_2_*CH_3_*), 1.65–1.74 (m, 2H, OCH_2_*CH_2_*CH_3_), 3.90 (t, *J* = 7.4 Hz, 2H, O*CH_2_*CH_2_CH_3_), 6.28 (s, 1H, H-3), 6.53 (d, *J* = 8.8 Hz, 1H, H-5’), 7.27–7.30 (m, 1H, H-6), 7.60–7.66 (m, 2H, H-7 and H-8), 7.87 (dd, *J* = 1.5 and 9.4 Hz, 1H, H-4’), 8.03 (d, *J* = 7.9 Hz, 1H, H-5), 8.38 (d, *J* = 2.5 Hz, 1H, H-2’), 11.45 ppm (s, 1H, OH).

#### 2-Pyridin-2-ylquinolin-4-ol (24)

General procedure B: time, 2 h; starting material, *N*-(2-acetylphenyl)pyridine-2-carboxamide **16** (0.50 g, 2.08 mmol). Compound **24**^24^ was obtained as a white solid (0.28 g, 61% yield), mp 236.5–237.0 °C. ^1^H NMR (200 MHz, DMSO-d_6_): *δ* = 6.83 (s, 1H, H-3), 7.27 (t, *J* = 6.8 Hz, 1H, H-6), 7.51–7.65 (m, 2H, H-7 and H-5’), 7.90–8.05 (m, 3H, H-8, H-3’ and H-4’), 8.22 (d, *J* = 7.5 Hz, 1H, H-5), 8.77 (d, *J* = 4.9 Hz, 1H, H-6’), 11.90 ppm (s, 1H, OH).

#### 2-Pyridin-3-ylquinolin-4-ol (25)

General procedure B: time, 7 h; starting material, *N*-(2-acetylphenyl)nicotinamide **17**^25^ (0.20 g, 0.83 mmol). Compound **25**^24^ was obtained as a yellow solid (0.13 g, 70% yield), mp 243.0–244.0 °C. ^1^H NMR (400 MHz, DMSO-d_6_): *δ* = 6.37 (s, 1H, H-3), 7.32 (t, *J* = 7.0 Hz, 1H, H-6), 7.58 (dd, *J* = 4.8 and 7.9 Hz, 1H, H-4’), 7.63–7.71 (m, 2H, H-7 and H-5’), 8.07 (d, *J* = 7.9 Hz, 1H, H-8), 8.20 (d, *J* = 7.9 Hz, 1H, H-5), 8.72 (d, *J* = 4.5 Hz, 1H, H-6’), 8.99 (d, *J* = 1.8 Hz, 1H, H-2’), 11.81 ppm (s, 1H, OH).

#### 2-Pyridin-4-ylquinolin-4-ol (26)

General procedure B: time, 8 h; starting material, *N*-(2-acetylphenyl)isonicotinamide**18**^26^ (1.00 g, 4.16 mmol). Compound **26**^24^ was obtained as a white solid (0.53 g, 57% yield), mp > 300 °C. ^1^H NMR (400 MHz, DMSO-d_6_): *δ* = 6.64 (s, 1H, H-3), 7.35 (t, *J* = 7.3 Hz, 1H, H-6), 7.68 (dt, *J* = 1.4 and 8.1 Hz, 1H, H-7), 7.76 (d, *J* = 8.2 Hz, 1H, H-8), 7.85 (d, *J* = 6.2 Hz, 2H, H-3’ and H-5’), 8.08 (dd, *J* = 1.3 and 8.1 Hz, 1H, H-5), 8.76 (d, *J* = 5.1 Hz, 2H, H-2’ and H-6’), 11.94 ppm (s, 1H, OH).

#### 2–(2-Thienyl)quinolin-4-ol (27)

General procedure B: time, 8 h; starting material, *N*-(2-acetylphenyl)thiophene-2-carboxamide **19**^27^ (0.20 g, 0.82 mmol). Compound **27**^24^ was obtained as a pink solid (0.11 g, 62% yield), mp > 300 °C. ^1^H NMR (400 MHz, DMSO-d_6_): *δ* = 6.43 (s, 1H, H-3), 7.23–7.25 (m, 1H, H-5’), 7.30 (t, *J* = 7.5 Hz, 1H, H-6), 7.63 (t, *J* = 7.1 Hz, 1H, H-7), 7.77–7.91 (m, 3H, H-8, H-3’ and H-4’), 8.03 (d, *J* = 8.0 Hz, 1H, H-5), 11.74 ppm (s, 1H, OH).

#### 2–(3-Thienyl)quinolin-4-ol (28)

General procedure B: time, 8 h; starting material, *N*-(2-acetylphenyl)thiophene-3-carboxamide **20** (0.20 g, 0.82 mmol). Compound **28**^24^ was obtained as a white solid (0.13 g, 72% yield), mp > 300 °C. ^1^H NMR (400 MHz, DMSO-d_6_): *δ* = 6.47 (s, 1H, H-3), 7.30 (t, *J* = 7.1 Hz, 1H, H-6), 7.63 (dd, *J* = 1.2 and 7.0 Hz, 1H, H-7), 7.68–7.77 (m, 3H, H-8, H-4’ and H-5’), 8.01 (d, *J* = 8.1 Hz, 1H, H-5), 8.24–8.31 (m, 1H, H-4’), 11.64 ppm (s, 1H, OH).

#### 2–(5-Chloro-2-thienyl)quinolin-4-ol (29)

General procedure B: time, 8 h; starting material, *N*-(2-acetylphenyl)-5-chlorothiophene-2-carboxamide **21** (0.50 g, 1.79 mmol). Compound **29** was obtained as a yellow solid (0.32 g, 73% yield), mp > 300 °C. ^1^H NMR (200 MHz, DMSO-d_6_): *δ* = 6.74 (s, 1H, H-3), 7.28 (d, *J* = 4.1 Hz, 1H, H-3’), 7.40 (t, *J* = 7.1 Hz, 1H, H-6), 7.70 (dt, *J* = 2.0 and 7.1 Hz, 1H, H-7), 7.81–7.92 (m, 2H, H-8 and H-4’), 8.06 (d, *J* = 8.2 Hz, 1H, H-5), 11.64 ppm (s, 1H, OH).

#### General procedure C for the synthesis of compounds 30a,b–37a,b

To a solution of derivatives **22–29** (1 equiv) in dry DMF (10 per mmol), K_2_CO_3_ (4 equiv) and 2-chloro-*N*,*N*-dimethylethylamine hydrochloride or 1–(2-chloroethyl)piperidine hydrochloride (3 equiv) were added. The reaction mixture was stirred at 80 °C for 2–12 h, then it was poured in ice/water and extracted with EtOAc. The organic layers were washed with brine, dried over Na_2_SO_4_ and evaporated under vacuum to give crude oils. After purification by flash chromatography column, compounds (**30b**, **32b**, **33b**, **34a**, **35a**, **36a**, **36b** and **37b**) were obtained as solids. Differently, to a solution of the compounds (**30a**, **31a**, **31b**, **32a**, **33a**, **34b**, **35b** and **37a**) in Et_2_O, HCl_gas_ was bubbled and, after filtration, compounds were collected as hydrochloride solids.

#### *N*,*N*-diethyl-2-{[2–(5-propoxypyridin-2-yl)quinolin-4-yl]oxy}ethanamine hydrochloride (30a)

General procedure C: time, 4 h; starting materials, 2–(5-propoxypyridin-2-yl)quinolin-4-ol **22** (0.20 g, 0.71 mmol) and (2-chloroethyl)diethylamine hydrochloride. After purification (CH_2_Cl_2_/MeOH 97/3) and hydrochlorination, compound **30a** was obtained as a white solid (0.04 g, 17% yield), mp 212.0–213.0 °C. ^1^H NMR (400 MHz, DMSO-d_6_): *δ* = 0.99 (t, *J* = 7.4 Hz, 3H, OCH_2_CH_2_*CH_3_*), 1.29 (t, *J* = 7.4 Hz, 6H, NCH_2_*CH_3_* x 2), 1.73–1.82 (m, 2H, OCH_2_*CH_2_*CH_3_), 3.21–3.28 (m, 4H, N*CH_2_*CH_3_ x 2), 3.71–3.75 (m, 2H, OCH_2_*CH_2_*N), 4.14 (t, *J* = 6.6 Hz, 2H, O*CH_2_*CH_2_CH_3_), 4.93–4.97 (m, 2H, O*CH_2_*CH_2_N), 7.70–7.75 (m, 2H, H-6 and H-4’), 7.97 (t, *J* = 7.7 Hz, 1H, H-7), 8.11 (s, 1H, H-3), 8.39–8.42 (m, 2H, H-8 and H-3’), 8.53 (d, *J* = 2.6 Hz, 1H, H-6’), 8.80 (d, *J* = 8.6 Hz, 1H, H-5), 11.32 ppm (s, 1H, HCl). ^13^C NMR (101 MHz, DMSO-d_6_): *δ* = 8.93, 10.74, 22.32, 47.32, 49.64, 66.26, 70.51, 99.93, 120.29, 122.01, 123.27, 125.30, 125.43, 125.57, 127.96, 127.99, 133.39, 133.72, 138.98, 154.37, 157.76 ppm. Anal calcd for C_23_H_30_ClN_3_O_2_: C, 66.41; H, 7.27; N, 10.10; found: C, 66.38; H, 7.28; N, 10.13.

#### 4–(2-Piperidin-1-ylethoxy)-2–(5-propoxypyridin-2-yl)quinoline (30b)

General procedure C: time, 4 h; starting materials, 2–(5-propoxypyridin-2-yl)quinolin-4-ol **22** (0.23 g, 0.81 mmol) and 1–(2-chloroethyl)piperidine hydrochloride. After purification (CH_2_Cl_2_/MeOH 95/5), compound **30b** was obtained as a white solid (0.11 g, 33% yield), mp 90.0–91.0 °C. ^1^H NMR (400 MHz, CDCl_3_): *δ* = 1.05 (t, *J* = 7.4 Hz, 3H, OCH_2_CH_2_*CH_3_*), 1.39–1.45 (m, 2H, piperidine CH_2_), 1.52–1.63 (m, 4H, piperidine CH_2_ x 2), 1.78–1.97 (m, 2H, OCH_2_*CH_2_*CH_3_), 2–49-2.61 (m, 4H, piperidine NCH_2_ × 2), 2.97 (t, *J* = 5.9 Hz, 2H, OCH_2_*CH_2_*N), 4.02 (t, *J* = 6.5 Hz, 2H, O*CH_2_*CH_2_CH_3_), 4.46 (t, *J* = 5.9 Hz, 2H, O*CH_2_*CH_2_N), 7.31 (dd, *J* = 2.8 and 8.7 Hz, 1H, H-4’), 7.45 (t, *J* = 7.3 Hz, 1H, H-6), 7.66 (t, *J* = 7.1 Hz, 1H, H-7), 7.89 (s, 1H, H-3), 8.03 (d, *J* = 8.2 Hz, 1H, H-8), 8.17 (d, *J* = 8.1 Hz, 1H, H-5), 8.36 (d, *J* = 2.5 Hz, 1H, H-6’), 8.56 ppm (d, *J* = 8.8 Hz, 1H, H-3’). ^13^C NMR (101 MHz, CDCl_3_): *δ* = 10.47, 22.51, 24.17, 26.07, 54.99, 57.63, 66.75, 69.96, 97.85, 121.09, 121.55, 121.91, 122.49, 125.34, 129.00, 129.72, 136.91, 148.95, 155.98, 157.37, 161.99 ppm. Anal calcd for C_24_H_29_N_3_O_2_: C, 73.63; H, 7.47; N, 10.73;found: C, 73.70; H, 7.46; N, 10.71.

#### *N,N*-diethyl-2-{[2–(6-propoxypyridin-3-yl)quinolin-4-yl]oxy}ethanamine hydrochloride (31a)

General procedure C: time, 2 h; starting materials, 2–(6-propoxypyridin-3-yl)quinolin-4-ol **23** (0.20 g, 0.71 mmol) and (2-chloroethyl)diethylamine hydrochloride. After purification (CH_2_Cl_2_/MeOH 95/5) and hydrochlorination, compound **31a** was obtained as a white solid (0.19 g, 65% yield), mp 188.0–190.0 °C. ^1^H NMR (400 MHz, DMSO-d_6_): *δ* = 0.89 (t, *J* = 7.6 Hz, 3H, OCH_2_CH_2_*CH_3_*), 1.29 (t, *J* = 7.1 Hz, 6H, NCH_2_*CH_3_* x 2), 1.72–1.82 (m, 2H, OCH_2_*CH_2_*CH_3_), 3.21–3.31 (m, 4H, N*CH_2_*CH_3_ x 2), 3.61–3.68 (m, 2H, OCH_2_*CH_2_*N), 4.00 (t, *J* = 7.6 Hz, 2H, O*CH_2_*CH_2_CH_3_), 4.87–5.01 (m, 2H, O*CH_2_*CH_2_N), 6.60 (d, *J* = 9.7 Hz, 1H, H-5’), 7.72 (t, *J* = 7.5 Hz, 1H, H-6), 7.77 (s, 1H, H-3), 7.98 (t, *J* = 7.8 Hz, 1H, H-7), 8.37 (d, *J* = 8.3 Hz, 1H, H-5), 8.41 (dd, *J* = 2.4 and 9.6 Hz, 1H, H-4’), 8.52–8.54 (m, 1H, H-8), 9.26 (s, 1H, H-2’), 11.16 ppm (s, 1H, HCl). ^13^C NMR (101 MHz, DMSO-d_6_): *δ* = 9.01, 11.25, 22.49, 47.58, 49.82, 51.31, 65.19, 100.03, 112.58, 119.61, 123.10, 123.56, 123.72, 127.45, 133.25, 138.72, 142.69, 143.02, 153.97, 161.44, 164.62 ppm. Anal calcd for C_23_H_30_ClN_3_O_2_: C, 66.41; H, 7.27; N, 10.10;found: C, 66.37; H, 7.30; N, 10.12.

#### 4–(2-Piperidin-1-ylethoxy)-2–(6-propoxypyridin-3-yl)quinoline hydrochloride (31b)

General procedure C: time, 5 h; starting materials, 2–(6-propoxypyridin-3-yl)quinolin-4-ol **23** (0.30 g, 1.09 mmol) and 1–(2-chloroethyl)piperidine hydrochloride. After purification (CH_2_Cl_2_/MeOH 99/1) and hydrochlorination, compound **31 b** was obtained as a white solid (0.25 g, 54% yield), mp 223.0–224.0 °C. ^1^H NMR (400 MHz, DMSO-d_6_): *δ* = 0.89 (t, *J* = 7.4 Hz, 3H, OCH_2_CH_2_*CH_3_*), 1.32–1.42 (m, 1H, piperidine CH), 1.67–1.90 (m, 7H, piperidine CH, piperidine CH_2_ x 2 and OCH_2_*CH_2_*CH_3_), 3.03–3.10 (m, 2H, piperidine NCH_2_), 3.37–3.40 (m, 2H, piperidine NCH_2_), 3.67–3.68 (m, 2H, OCH_2_*CH_2_*N), 3.99 (t, *J* = 8.1 Hz, 2H, O*CH_2_*CH_2_CH_3_), 4.95–5.00 (m, 2H, O*CH_2_*CH_2_N), 6.60 (d, *J* = 9.2 Hz, 1H, H-5’), 7.71 (t, *J* = 7.4 Hz, 1H, H-6), 7.75 (s, 1H, H-3), 7.97 (t, *J* = 7.5 Hz, 1H, H-7), 8.40–8.43 (m, 2H, H-8 and H-4’), 8.45–8.51 (m, 1H, H-5), 9.22 (s, 1H, H-2’), 11.19 ppm (s, 1H, HCl). ^13^C NMR (101 MHz, DMSO-d_6_): *δ* = 11.25, 21.63, 22.50, 22.76, 51.30, 52.89, 54.49, 64.87, 99.72, 119.64, 123.10, 124.06, 124.26, 127.26, 132.97, 133.15, 138.69, 142.37, 142.59, 154.07, 161.47, 164.12. Anal calcd for C_24_H_30_ClN_3_O_2_: C, 67.36; H, 7.07; N, 9.82;found: C, 67.42; H, 7.06; N, 9.80.

#### *N*,*N*-diethyl-2-[(2-pyridin-2-ylquinolin-4-yl)oxy]ethanamine hydrochloride (32a)

General procedure C: time, 12 h; starting materials, 2-pyridin-2-ylquinolin-4-ol **24** (0.30 g, 1.35 mmol) and (2-chloroethyl)diethylamine hydrochloride. After purification (CH_2_Cl_2_/MeOH 95/5) and hydrochlorination, compound **32a** was obtained as a white solid (0.10 g, 21% yield), mp 173.0–174.0 °C. ^1^H NMR (400 MHz, DMSO-d_6_): *δ* = 1.28 (t, *J* = 7.2 Hz, 6H, NCH_2_*CH_3_* x 2), 3.21–3.29 (m, 4H, N*CH_2_*CH_3_ x 2), 3.67–3.71 (m, 2H, OCH_2_*CH_2_*N), 4.76 (t, *J* = 4.9 Hz, 2H, O*CH_2_*CH_2_N), 7.52 (ddd, *J* = 1.1, 4.8 and 9.1 Hz, 1H, H-3’), 7.60 (dt, *J* = 1.1 and 7.0 Hz, 1H, H-6), 7.79 (dt, *J* = 1.4 and 7.0, 1H, H-7), 7.99 (dt, *J* = 1.8 and 8.0 Hz, 1H, H-5’), 7.81–8.06 (m, 2H, H-3 and H-4’), 8.25 (dd, *J* = 1.0 and 8.2 Hz, 1H, H-8), 8.59 (d, *J* = 7.9 Hz, 1H, H-5), 8.73 (dd, *J* = 1.5 and 5.0 Hz, 1H, H-6’), 10.70 ppm (s, 1H, HCl). ^13^C NMR (101 MHz, DMSO-d_6_): *δ* = 9.06, 47.52, 49.92, 63.66, 120.90, 121.72, 122.35, 125.41, 126.91, 129.29, 130.99, 137.94, 148.52, 149.58, 155.25, 156.97, 161.51 ppm. Anal calcd for C_20_H_24_ClN_3_O: C, 67.12; H, 6.76; N, 11.74;found: C, 67.21; H, 6.76; N, 11.70.

#### 4–(2-Piperidin-1-ylethoxy)-2-pyridin-2-ylquinoline (32b)

General procedure C: time, 4 h; starting materials, 2-pyridin-2-ylquinolin-4-ol **24** (0.21 g, 0.93 mmol) and 1–(2-chloroethyl)piperidine hydrochloride. After purification (CHCl_3_/MeOH 97/3), compound **32 b** was obtained as a white solid (0.21 g, 69% yield), mp 77.0–79.5 °C. ^1^H NMR (400 MHz, DMSO-d_6_): *δ* = 1.33–1.35 (m, 2H, piperidine CH_2_), 1.44–1.50 (m, 4H, piperidine CH_2_ x 2), 2.46–2.49 (m, 4H, piperidine NCH_2_ × 2), 2.84 (t, *J* = 6.4 Hz, 2H, OCH_2_*CH_2_*N), 4.41 (t, *J* = 6.8 Hz, 2H, O*CH_2_*CH_2_N), 7.48 (ddd, *J* = 1.2, 5.1 and 8.9 Hz, 1H, H-3’), 7.55 (dt, *J* = 1.9 and 7.6 Hz, 1H, H-6), 7.75 (dt, *J* = 1.8 and 7.3 Hz, 1H, H-7), 7.97 (dt, *J* = 2.5 and 8.4 Hz, 1H, H-5’), 7.99–8.01 (m, 2H, H-3 and H-4’), 8.11 (d, *J* = 7.9 Hz, 1H, H-8), 8.56 (dd, *J* = 2.0 and 7.9 Hz, 1H, H-5), 8.70 ppm (dd, *J* = 1.2 and 5.4 Hz, 1H, H-6’). ^13^C NMR (101 MHz, DMSO-d_6_): *δ* = 24.30, 26.07, 54.79, 57.41, 67.17, 98.46, 121.19, 121.60, 122.01, 125.19, 126.67, 129.44, 130.69, 137.77, 148.71, 149.57, 155.66, 157.19, 162.04 ppm. Anal calcd for C_21_H_23_N_3_O: C, 75.65; H, 6.95; N, 12.60;found: C, 75.74; H, 6.93; N, 12.54.

#### *N*,*N*-diethyl-2-[(2-pyridin-3-ylquinolin-4-yl)oxy]ethanamine hydrochloride (33a)

General procedure C: time, 5 h; starting materials, 2-pyridin-3-ylquinolin-4-ol **25** (0.30 g, 1.35 mmol) and (2-chloroethyl)diethylamine hydrochloride. After purification (CH_2_Cl_2_/MeOH 90/10) and hydrochlorination, compound **33a** was obtained as a white solid (0.17 g, 35% yield), mp 181.0–182.0 °C. ^1^H NMR (400 MHz, DMSO-d_6_): *δ* = 1.29 (t, *J* = 7.1 Hz, 6H, NCH_2_*CH_3_* x 2), 3.12–3.25 (m, 4H, N*CH_2_*CH_3_ × 2), 3.70–3.71 (m, 2H, OCH_2_*CH_2_*N), 4.90–4.93 (m, 2H, O*CH_2_*CH_2_N), 7.71 (t, *J* = 7.5 Hz, 1H, H-6), 7.91 (t, *J* = 7.7 Hz, 1H, H-7), 7.98 (s, 1H, H-3), 8.09 (t, *J* = 6.1 Hz, 1H, H-5’), 8.25 (d, *J* = 8.3 Hz, 1H, H-8), 8.35 (d, *J* = 8.2 Hz, 1H, H-5), 8.98 (d, *J* = 7.9 Hz, 1H, H-6’), 9.26 (d, *J* = 8.1 Hz, 1H, H-4’), 9.68 (s, 1H, H-2’), 11.31 ppm (s, 1H, HCl). ^13^C NMR (101 MHz, DMSO-d_6_): *δ* = 24.30, 26.07, 54.79, 57.41, 67.17, 98.46, 121.19, 121.60, 122.01, 125.19, 126.67, 129.44, 130.69, 137.77, 148.71, 149.57, 155.66, 157.19, 162.04 ppm. Anal calcd for C_20_H_24_ClN_3_O: C, 67.12; H, 6.76; N, 11.74;found: C, 67.31; H, 6.75; N, 11.70.

#### 4–(2-Piperidin-1-ylethoxy)-2-pyridin-3-ylquinoline (33b)

General procedure C: time, 4 h; starting materials, 2-pyridin-3-ylquinolin-4-ol **25** (0.30 g, 1.35 mmol) and 1–(2-chloroethyl)piperidine hydrochloride. After purification (CH_2_Cl_2_/MeOH 95/5), compound **33 b** was obtained as a white solid (0.26 g, 58% yield), mp 101.5–102.5 °C. ^1^H NMR (400 MHz, DMSO-d_6_): *δ* = 1.33–1.36 (m, 2H, piperidine CH_2_), 1.47–1.52 (m, 4H, piperidine CH_2_ x 2), 2.47–2.51 (m, 4H, piperidine NCH_2_ x 2), 2.81–2.85 (m, 2H, OCH_2_*CH_2_*N), 4.47 (t, *J* = 5.8 Hz, 1H, O*CH_2_*CH_2_N), 7.52–7.58 (m, 2H, H-6 and H-5’), 7.64 (s, 1H, H-3), 7.74 (t, *J* = 7.4 Hz, 1H, H-7), 7.99 (d, *J* = 8.4 Hz, 1H, H-8), 8.10 (d, *J* = 8.2 Hz, 1H, H-5), 8.61 (d, *J* = 7.9 Hz, 1H, H-6’), 8.65 (d, *J* = 4.7 Hz, 1H, H-4’), 9.43 ppm (s, 1H, H-2’). ^13^C NMR (101 MHz, DMSO-d_6_): *δ* = 24.29, 26.05, 54.76, 57.50, 67.37, 99.59, 120.49, 121.97, 124.13, 126.39, 129.36, 130.73, 134.96, 135.17, 148.94, 149.06, 150.73, 155.87, 162.26 ppm. Anal calcd for C_21_H_23_N_3_O: C, 75.65; H, 6.95; N, 12.60;found: C, 75.71; H, 6.93; N, 12.57.

#### *N*,*N*-diethyl-2-[(2-pyridin-4-ylquinolin-4-yl)oxy]ethanamine (34a)

General procedure C: time, 4 h; starting materials, 2-pyridin-4-ylquinolin-4-ol **26** (0.30 g, 1.35 mmol) and (2-chloroethyl)diethylamine hydrochloride. After purification (CH_2_Cl_2_/MeOH 90/10), compound **34a** was obtained as a white solid (0.22 g, 51% yield), mp 73.5–74.0 °C. ^1^H NMR (400 MHz, CDCl_3_): *δ* = 1.09 (t, *J* = 6.1 Hz, 6H, NCH_2_*CH_3_* x 2), 2.70 (q, *J* = 7.3 Hz, 4H, N*CH_2_*CH_3_ x 2), 3.05 (t, *J* = 6.0 Hz, 2H, OCH_2_*CH_2_*N), 4.34 (t, *J* = 6.2 Hz, 2H, O*CH_2_*CH_2_N), 7.19 (s, 1H, H-3), 7.50 (t, *J* = 7.2 Hz, 1H, H-6), 7.71 (t, *J* = 6.8 Hz, 1H, H-7), 7.97–7.99 (m, 2H, H-3’ and H-5’), 8.08 (d, *J* = 8.9 Hz, 1H, H-8), 8.18 (d, *J* = 8.3 Hz, 1H, H-5), 8.71–8.77 ppm (m, 2H, H-2’ and H-6’). ^13^C NMR (101 MHz, CDCl_3_): *δ* = 12.08, 48.06, 51.40, 67.68, 98.19, 120.92, 121.66, 121.80, 126.15, 129.43, 130.29, 147.29, 149.20, 150.41, 155.83, 162.52 ppm. Anal calcd for C_20_H_23_N_3_O: C, 74.74; H, 7.21; N, 13.07;found: C, 74.81; H, 7.19; N, 13.01.

#### 4–(2-Piperidin-1-ylethoxy)-2-pyridin-4-ylquinoline hydrochloride (34b)

General procedure C: time, 4 h; 2-pyridin-4-ylquinolin-4-ol **26** (0.30 g, 1.35 mmol) and 1–(2-chloroethyl)piperidine hydrochloride. After purification (CH_2_Cl_2_/MeOH 99/1) and hydrochlorination, compound **34 b** was obtained as a white solid (0.30 g, 61% yield), mp 183.5–185.0 °C. ^1^H NMR (400 MHz, DMSO-d_6_): *δ* = 1.33–1.42 (m, 2H, piperidine CH_2_), 1.66–1.93 (m, 4H, piperidine CH_2_ × 2), 3.04–3.09 (m, 2H, piperidine NCH_2_), 3.51–3.54 (m, 2H, piperidine NCH_2_), 3.63–3.67 (m, 2H, OCH_2_*CH_2_*N), 4.93 (t, *J* = 5.0 Hz, 2H, O*CH_2_*CH_2_N), 7.69 (t, *J* = 7.3 Hz, 1H, H-6), 7.87 (t, *J* = 7.0 Hz, 1H, H-7), 8.02 (s, 1H, H-3), 8.15 (d, *J* = 8.4 Hz, 1H, H-8), 8.35 (d, *J* = 8.1 Hz, 1H, H-5), 8.92 (d, *J* = 6.7 Hz, 2H, H-3’ and H-5’), 9.08 (d, *J* = 6.7 Hz, 2H, H-2’ and H-6’), 11.42 ppm (s, 1H, HCl). ^13^C NMR (101 MHz, DMSO-d_6_): *δ* = 21.66, 22.72, 52.74, 54.52, 64.34, 101.11, 121.09, 122.73, 124.99, 128.38, 129.29, 131.92, 143.00, 148.19, 152.38, 153.36, 162.55 ppm. Anal calcd for C_21_H_24_ClN_3_O: C, 68.19; H, 6.54; N, 11.36;found: C, 68.01; H, 6.56; N, 11.40.

#### *N*,*N*-diethyl-2-{[2–(2-thienyl)quinolin-4-yl]oxy}ethanamine (35a)

General procedure C: time, 4 h; starting materials, 2–(2-thienyl)quinolin-4-ol **27** (0.30 g, 1.32 mmol) and (2-chloroethyl)diethylamine hydrochloride. After purification (CH_2_Cl_2_/MeOH 98/2), compound **35a** was obtained as a white solid (0.26 g, 60% yield), mp 84.0–86.0 °C. ^1^H NMR (400 MHz, CDCl_3_): *δ* = 0.99 (t, *J* = 7.0 Hz, 6H, NCH_2_*CH_3_* × 2), 2.58 (q, *J* = 7.0 Hz, 4H, N*CH_2_*CH_3_ x 2), 2.93 (t, *J* = 5.5 Hz, 2H, OCH_2_*CH_2_*N), 4.37 (t, *J* = 5.4 Hz, 2H, O*CH_2_*CH_2_N), 7.17–7.19 (m, 1H, H-4’), 7.47 (t, *J* = 7.3 Hz, 1H, H-6), 7.54 (s, 1H, H-3), 7.65–7.69 (m, 2H, H-7 and H-5’), 7.84 (d, *J* = 8.4 Hz, 1H, H-8), 8.02–8.04 ppm (m, 2H, H-5 and H-3’). ^13^C NMR (101 MHz, CDCl_3_): *δ* = 12.50, 47.53, 51.37, 68.08, 98.05, 120.52, 121.94, 125.77, 127.52, 128.61, 128.68, 129.89, 130.70, 145.74, 148.71, 153.64, 161.91 ppm. Anal calcd for C_19_H_22_N_2_OS: C, 69.90; H, 6.79; N, 8.58;found: C, 69.99; H, 6.77; N, 8.58.

#### 4–(2-piperidin-1-ylethoxy)-2–(2-thienyl)quinoline hydrochloride (35b)

General procedure C: time, 5 h; 2–(2-thienyl)quinolin-4-ol **27** (0.30 g, 1.32 mmol) and 1–(2-chloroethyl)piperidine hydrochloride. After purification (CH_2_Cl_2_/MeOH 95/5) and hydrochlorination, compound **35 b** was obtained as a white solid (0.29 g, 58% yield), mp 208.0–209.5 °C. ^1^H NMR (400 MHz, DMSO-d_6_): *δ* = 1.66–1.69 (m, 2H, piperidine CH_2_), 1.76–1.89 (m, 4H, piperidine CH_2_ × 2), 3.01–3.14 (m, 2H, piperidine NCH_2_), 3.42–3.55 (m, 2H, piperidine NCH_2_), 3.62–3.71 (m, 2H, OCH_2_*CH_2_*N), 4.87–4.89 (m, 2H, O*CH_2_*CH_2_N), 7.28 (t, *J =* 4.1 Hz, 1H, H-4’), 7.59 (t, *J* = 7.4 Hz, 1H, H-6), 7.62 (s, 1H, H-3), 7.83 (t, *J* = 7.6 Hz, 1H, H-7), 7.88 (d, *J* = 4.5 Hz, 1H, H-5’), 8.16 (d, *J* = 6.9 Hz, 1H, H-8), 8.30 (d, *J* = 8.2 Hz, 1H, H-5), 8.34 (bs, 1H, H-3’), 11.33 ppm (s, 1H, HCl). ^13^C NMR (101 MHz, DMSO-d_6_): *δ* = 21.61, 22.81, 52.82, 54.64, 64.21, 99.15, 120.07, 122.80, 126.68, 129.13, 129.63, 130.14, 131.68, 132.03, 144.46, 146.35, 152.64, 162.50 ppm. Anal calcd for C_20_H_23_ClN_2_OS: C, 64.07; H, 6.18; N, 7.47;found: C, 64.11; H, 6.17; N, 7.45.

#### *N*,*N*-diethyl-2-{[2–(3-thienyl)quinolin-4-yl]oxy}ethanamine (36a)

General procedure C: time, 4 h; starting materials, 2–(3-thienyl)quinolin-4-ol **28** (0.30 g, 1.32 mmol) and (2-chloroethyl)diethylamine hydrochloride. After purification (CH_2_Cl_2_/MeOH 95/5), compound **36a** was obtained as a white solid (0.13 g, 30% yield), mp 66.0–67.0 °C. ^1^H NMR (400 MHz, CDCl_3_): *δ* = 1.12 (t, *J* = 7.3 Hz, 6H, NCH_2_*CH_3_* × 2), 2.71 (q, *J* = 7.0 Hz, 4H, N*CH_2_*CH_3_ × 2), 3.07 (t, *J* = 6.1 Hz, 2H, OCH_2_*CH_2_*N), 4.34 (t, *J* = 6.1 Hz, 2H, O*CH_2_*CH_2_N), 7.09 (s, 1H, H-3), 7.39–7.47 (m, 2H, H-6 and H-8), 7.66 (dt, *J* = 1.4 and 8.4 Hz, 1H, H-7), 7.80 (dd, *J* = 1.3 and 5.0 Hz, 1H, H-5’), 7.98 (dd, *J* = 1.1 and 4.4 Hz, 1H, H-4’), 8.02 (d, *J* = 8.5 Hz, 1H, H-5), 8.12 ppm (dd, *J* = 1.1 and 8.3 Hz, 1H, H-2’). ^13^C NMR (101 MHz, CDCl_3_): *δ* = 11.92, 47.48, 51.29, 67.16, 98.55, 120.39, 121.64, 124.44, 125.18, 126.27, 126.85, 128.95, 129.96, 143.12, 149.19, 154.51, 161.85 ppm. Anal calcd for C_19_H_22_N_2_OS: C, 69.90; H, 6.79; N, 8.58;found: C, 69.94; H, 6.78; N, 8.56.

#### 4–(2-Piperidin-1-ylethoxy)-2–(3-thienyl)quinoline (36b)

General procedure C: time, 3 h; starting materials, 2–(3-thienyl)quinolin-4-ol **28** (0.30 g, 1.32 mmol) and 1–(2-chloroethyl)piperidine hydrochloride. After purification (CH_2_Cl_2_/MeOH 98/2), compound **36b** was obtained as a white solid (0.27 g, 61% yield), mp 109.5–111.0 °C. ^1^H NMR (400 MHz, CDCl_3_): *δ* = 1.44–1.48 (m, 2H, piperidine CH_2_), 1.60–1.66 (m, 4H, piperidine CH_2_ × 2), 2.55–2.61 (m, 4H, piperidine CH_2_ × 2), 2.98 (t, *J* = 6.8 Hz, 2H, OCH_2_*CH_2_*N), 4.40 (t, *J* = 6.0 Hz, 2H, O*CH_2_*CH_2_N), 7.08 (s, 1H, H-3), 7.40–7.48 (m, 2H, H-6 and H-8), 7.66 (dt, *J* = 1.5 and 8.3 Hz, 1H, H-7), 7.80 (dd, *J* = 1.4 and 5.0 Hz, 1H, H-5’), 7.97 (dd, *J* = 1.1 and 4.2 Hz, 1H, H-4’), 8.01 (d, *J* = 8.2 Hz, 1H, H-5), 8.13 ppm (dd, *J* = 1.1 and 8.3 Hz, 1H, H-2’). ^13^C NMR (101 MHz, CDCl_3_): *δ* = 24.01, 25.94, 55.13, 57.52, 66.67, 98.55, 120.41, 121.68, 124.43, 125.18, 126.27, 126.85, 128.94, 129.97, 143.12, 149.18, 154.52, 161.78 ppm. Anal calcd for C_20_H_22_N_2_OS: C, 70.97; H, 6.55; N, 8.28;found: C, 70.85; H, 6.56; N, 8.30.

#### (2-{[2–(5-Chloro-2-thienyl)quinolin-4-yl]oxy}ethyl)diethylamine hydrochloride (37a)

General procedure C: time, 4 h; starting materials, 2–(5-chloro-2-thienyl)quinolin-4-ol **29** (1.00 g, 4.14 mmol) and (2-chloroethyl)diethylamine hydrochloride. After purification (CH_2_Cl_2_/MeOH 95/5) and hydrochlorination, compound **37a** was obtained as a white solid (0.50 g, 32% yield), mp 189.0–191.0 °C. ^1^H NMR (400 MHz, DMSO-d_6_): *δ* = 1.28 (t, *J* = 7.2 Hz, 6H, NCH_2_*CH_3_* × 2), 3.19–3.26 (m, 4H, N*CH_2_*CH_3_ × 2), 3.65–3.83 (m, 2H, OCH_2_*CH_2_*N), 4.76–4.78 (m, 2H, O*CH_2_*CH_2_N), 9.76 (d, *J* = 4.1 Hz, 1H, H-3’), 7.54 (t, *J* = 7.0 Hz, 1H, H-6), 7.63 (s, 1H, H-3), 7.74 (dt, *J* = 1.1 and 7.0 Hz, 1H, H-7), 7.92 (d, *J* = 8.3 Hz, 1H, H-8), 8.05 (d, *J* = 4.0 Hz, 1H, H-4’), 8.18 (d, *J* = 8.1 Hz, 1H, H-5), 11.07 ppm (s, 1H, HCl). ^13^C NMR (101 MHz, DMSO-d_6_): *δ* = 9.01, 47.40, 49.68, 64.01, 98.10, 120.29, 122.47, 126.48, 127.91, 128.10, 128.81, 131.44, 132.64, 141.11, 145.34, 152.34, 161.72 ppm. Anal calcd for C_19_H_22_Cl_2_N_2_OS: C, 57.43; H, 5.58; N, 7.05;found: C, 57.50; H, 5.56; N, 7.04.

#### 2–(5-Chloro-2-thienyl)-4–(2-piperidin-1-ylethoxy)quinoline (37b)

General procedure C: time, 3 h; starting materials, 2–(5-chloro-2-thienyl)quinolin-4-ol **29** (0.29 g, 1.20 mmol) and 1–(2-chloroethyl)piperidine hydrochloride. After purification (CH_2_Cl_2_/MeOH 95/5), compound **37b** was obtained as a white solid (0.14 g, 35% yield), mp 99.0–101.0 °C. ^1^H NMR (400 MHz, CDCl_3_): *δ* = 1.47–1.51 (m, 2H, piperidine CH_2_), 1.66–1.71 (m, 4H, piperidine CH_2_ × 2), 2.58–2.65 (m, 4H, piperidine NCH_2_ × 2), 3.03 (t, *J* = 5.7 Hz, 2H, OCH_2_*CH_2_*N), 4.44 (t, *J* = 5.7 Hz, 2H, O*CH_2_*CH_2_N), 6.92 (d, *J* = 4.0 Hz, 1H, H-3’), 7.01 (s, 1H, H-3), 7.39–7.43 (m, 2H, H-6 and H-4’), 7.64 (dt, *J* = 1.5 and 8.6 Hz, 1H, H-7), 7.94 (d, *J* = 8.2 Hz, 1H, H-8), 8.07 ppm (d, *J* = 7.3 Hz, 1H, H-5). ^13^C NMR (101 MHz, CDCl_3_): *δ* = 23.72, 25.52, 55.00, 57.27, 66.34, 93.14, 120.55, 121.62, 124.45, 125.40, 127.07, 128.72, 130.22, 133.31, 144.40, 148.91, 152.48, 161.60 ppm. Anal calcd for C_20_H_21_ClN_2_OS: C, 64.42; H, 5.68; N, 7.51;found: C, 64.50; H, 5.66; N, 7.50.

### Microbiological procedures

The strains of *S. aureus* used in this study included SA-1199B, which overexpresses *norA* and possesses an A116E GrlA substitution, and its isogenic parent SA-1199 (*norA* wt). MICs assays were performed using broth microdilution method in 96 wells-microtiter plates, following the CLSI guidelines^28^.

Chequerboard assays and time-kill curves were performed as previously described^29^. The formers were performed using 2-fold increasing concentrations of both antibiotic (from 0.001 to 20 µg/mL) and compounds (from 0.39 to 25 µg/mL), while time-kill curves were performed testing CPX concentrations ranging from ¼x to 1x MIC alone and in combination with the compound **37a** at 3.13 and 6.25 µg/mL. The dynamic of the bactericidal activity of the combination CPX-Compound was evaluated by CFU counts after 2, 4, 6, 8 and 24 h incubation at 37 °C.

FIC values for **37a** and CPX were calculated according to the following equation: MIC in combination/MIC alone. FICI values were calculated by the sum of FIC values of **37a** and CPX.

The loss of EtBr from *S. aureus* SA-1199B was determined fluorometrically as previously described^30^. The effect of various concentrations of tested compounds on the EtBr efflux of SA-1199B was compared to that in their absence, allowing the calculation of the percentage reduction in efflux.

Haemolysis assays were performed as previously described^31^.

Cell viability assays for compounds **1** and **37a** were performed on human leukemic monocyte cell line (THP-1). THP-1 cells were grown in RPMI 1640 supplemented with 10% heat-inactivated foetal calf serum, 10,000 units of penicillin, and 10 µg of streptomycin/mL overnight to confluence. Monolayers were treated for 24 h at 37 °C with scalar concentration of tested compounds (0 − 250 µg/mL). Cell viability was then evaluated using an ATP bioluminescence kit (Via Light kit; Cambrex). Results are expressed as 50% cytotoxic concentration (CC_50_). The CC_50_ was defined as the concentration required to reduce the cell number by 50% compared to that for the untreated controls. Each concentration was tested in triplicate.

Membrane potential assays were carried out by measuring the effect of **1** and **37a** on the membrane potential using the BacLight Bacterial Membrane Potential Kit (Molecular Probes, Life Technologies) according to the manufacturer’s instructions. Briefly, SA-1199B was grown in MHB at 37 °C until reaching an OD600 of 0.6. Bacterial cells were then washed in PBS and diluted to 1 × 10^6^ CFU/mL with filtered PBS (filter 0.22 µm) in flow cytometry tubes. Ten microliters of 3 mM 3,3-diethyloxacarbocyanine iodide (DiOC_2_(3) in DMSO) was added to each tube (final concentration 30 µM) and mixed. Then, **1** or **37a** from a stock solution in DMSO (10 mg/mL) was added to reach final concentrations of 1, 5, and 10 µg/mL. As positive control, 10 µL of 500 µM carbonyl cyanide m-chlorophenylhydrazone (CCCP, final concentration 5 µM) was used to eradicate the proton gradient by eliminating the membrane potential. The samples were analysed after 30 min by measuring the fluorescence using a cytometer Attune NxT (ThermoFisher Scientific) with a laser emitting at 488 nm and collecting in the green and red channels. The red to green fluorescence ratio was determined and normalised against the emission from the DiOC_2_(3) blank tube having 1 ml of the bacterial suspension and a final concentration of DiOC_2_(3) of 30 µM. The results are presented as the percentage of depolarised membranes compared with the drug-free control.

### In silico ADME

Quinoline derivatives **1**, **2** and **37a** were built using the Schrodinger Maestro Interface^32^ and then imported in MetaSite^33^. The protonation state of the compounds was normalised at a pH of 7.5. The MetaSite analysis was performed using the liver enzymes model. Compond **37a** was built in SeeSAR^34^ which automatically assigns the proper geometry, protonation state and tautomeric form of the ligands using ProToss method^35^. The Optibrium models for PK properties predictions were downloaded from Optibrium webpage^36^.

## Results and discussion

### Chemistry

**Scheme 1.**
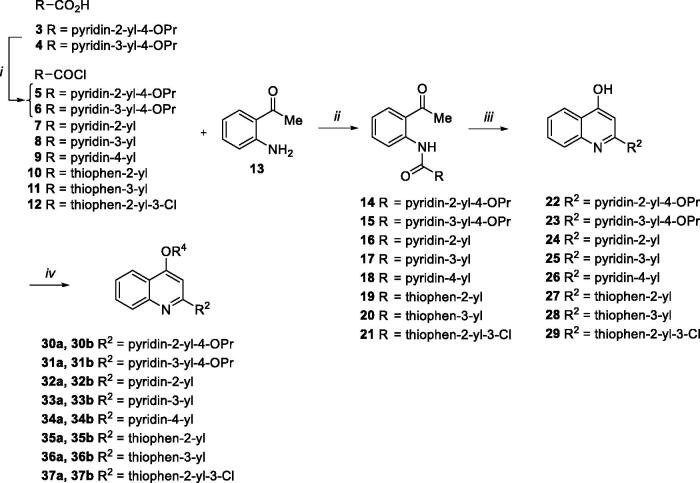
(i) SOCl_2_, 60 °C, 30 min; (ii) Et_3_N, dry THF, rt, 90 min-6 h, 50–99%; (iii) NaOH, dry dioxane, 110 °C, 2–8 h, 57–100%; (iv) chloroalkylamine hydrochloride, K_2_CO_3_, dry DMF, 80 °C, 2–12 h, 17–69%.

All designed compounds **30a**,**b**–**37a**,**b** were synthesised according to the procedure depicted in Scheme 1. Chlorination of acids **3** and **4** with SOCl_2_ afforded acyl chlorides **5** and **6** that were immediately reacted with the commercially available aminoacetophenone **13** in presence of Et_3_N to give the amide derivatives **14** and **15**. Similarly, coupling reaction of commercially available acyl chlorides **7**–**12** with **13** afforded amides **16–20**^24^ and **21**. Following the same procedure reported by Brouwer et al.^37^, ring closure of **14**–**21** through NaOH in dioxane at 110 °C in a pressure tube gave the C-2 aryl quinoline derivatives **22**, **23**, **24–28**^24^ and **29**. *O*-alkylation of 4-hydroxyquinolines **22**–**29** with 2-chloro-N,N-dimethylethylamine hydrochloride or 1–(2-chloroethyl)piperidine hydrochloride afforded desired quinoline analogues **30a**,**b**–**37a**,**b** (Scheme 1).

### Synergistic assays

In order to quickly identify derivatives having significant synergistic activity, the 16 compounds were firstly assayed at 25 µg/mL in combination with scalar concentrations of CPX against the *norA* overexpressing *S. aureus* strain SA-1199B (*norA+*/GrlA mutation)^38^ (data not shown). Four compounds (**30a**, **30b**, **35b** and **36b**) showed a significant synergistic effect producing a 4-fold reduction of the antibiotic MIC while derivative **37a** exhibited an impressive CPX MIC reduction (32-fold). Worthy of note, among the five active compounds, three were thiophene derivatives (**35b**, **36b** and **37a**) displaying their activity regardless of the position of the sulphur atom on the thiophene ring. On the contrary, only pyridine-2-yl-5-OPr derivatives (**30a** and **30b**) exhibited a significant synergistic effect whereas all the remaining pyridine analogues (**31a**, **31b**, **32a**, **32b**, **33a**, **33b**, **34a** and **34b**) lost EPI activity regardless of the nitrogen atom position within the pyridine moiety.

### Chequerboard assays

**Figure 2.**
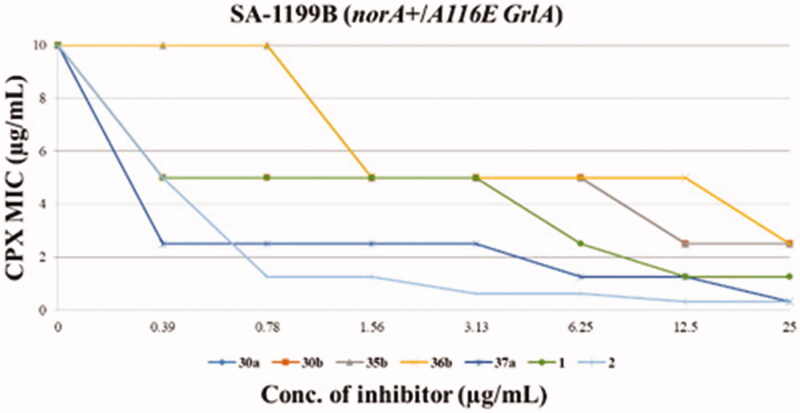
Chequerboard assays of compounds **30a**, **30b**, **35b**, **36b**, **37a** and reference compounds **1** and **2** in combination with CPX against SA-1199B.

The five derivatives showing the best synergistic activity were tested by chequerboard assays in combination with CPX against *S. aureus* SA-1199B (Figure 2), also including reference compounds **1** and **2**. The two quinoline analogues (**35b** and **36b**) with the C-2 unsubstituted thiophene portion did not retain any synergistic effect with CPX when their concentration did fall below 25 µg/mL. On the other hand, pyridine derivatives **30a** and **30b** at concentrations of 6.25 and 12.5 µg/mL, respectively, reduced the CPX MIC by 4-fold, thereby retaining an EPI activity comparable to **1**. Interestingly, the chlorothiophene derivative **37a** showed the best results being able, up to 0.39 µg/mL, to decrease by 4-fold the CPX MIC against SA-1199B, resulting about 16-fold more active than the starting hit **1**. Actually, compound **37a** exhibited a synergistic activity with CPX greater than that shown by compound **2**, which produced a weaker and less significant effect (2-fold MIC reduction) at the same concentration (0.39 µg/mL).

Thus, when rationalising SAR information, it appears clear as the replacement of the phenyl portion at quinoline C-2 with a thiophene ring was well tolerated when both *O*-ethylamino chains were present at the quinoline C-4 position. Interestingly, in the case of derivative **37a**, the substitution of the H-bond acceptor –*O*Pr group with a chlorine atom yielded very good results, paving the way for future modifications. On the contrary, the replacement of the phenyl portion at the quinoline C-2 position with pyridine moieties, regardless the presence of –OPr group, afforded less potent EPI derivatives. **30a** and **30b** compounds, when tested at the concentration of 25 µg/mL (synergistic assays) showed a potent synergistic effect with CPX while at lower concentrations (chequerboard assays), they displayed a poor ability to decrease the CPX MIC.

Focussing on the best identified compound **37a**, to indirectly rule out that its synergism with CPX against SA-1199B was due to non-specific effects, this derivative was tested at 25 µg/mL (a concentration 64-fold higher than that needed to reduce by 4-fold CPX MIC) in combination with scalar concentrations of CPX against the *S. aureus* strain SA-1199 (*norA* wild-type). Interestingly, compound **37a** did not exhibit any significant synergistic effect with CPX in presence of a *norA* basal expression (data not shown). Whether the synergistic activity of **37a** with CPX was due to a mechanism different from that of the NorA inhibition, we would have likely to observe a synergistic effect with CPX also against SA-1199 wild-type strain. Consequently, the observed high difference in the synergistic activity of **37a** with CPX against SA-1199B and SA-1199 strongly supports a **37a**-mediated NorA inhibition.

### MIC evaluation

Considering the very promising results of derivative **37a**, it was tested alone against SA-1199B in order to prove that the synergistic activity with CPX was not influenced by a direct antibacterial effect. Worthy of note, up to 25 µg/mL, this compound did not show any antibacterial activity. This result is essential for a potential EPI compound; indeed, a poor or absent antibacterial effect is a key requirement to avoid the evolutionary pressure on microorganisms that can evolve resistance only towards compounds endowed with an antimicrobial activity^39^. In this case, compound **37a** reduces CPX MIC (from 10 µg/mL to 2.5 µg/mL) at a concentration ≥64-fold lower than its MIC. As a confirmation of the synergistic effect of **37a** with CPX, we calculated the FIC values for both compounds: FIC**_37a_** < 0.0156 and FIC_CPX_ = 0.25. The sum of both FIC values led to a FIC index (FICI) < 0.27 showing a significant synergistic effect as widely recognised for FICI values <0.5^40^. Therefore, although FICI evaluation is mainly used to determine the synergism between two antimicrobial agents, the EPI **37a**, devoid of any antibacterial effect, exhibited a clear synergistic activity with the fluoroquinolone CPX.

### EtBr efflux assays

Since EtBr is a known NorA substrate resulting fluorescent only when inside bacterial cells, monitoring bacterial fluorescence of SA-1199B, overexpressing *norA* gene, through a fluorimeter is a fast method to indirectly evaluate NorA inhibition. Thus, to demonstrate that the synergistic effect of **37a** with CPX was due to the NorA efflux inhibition, we performed EtBr efflux assays against SA-1199B. Interestingly, at 50 µM compound **37a** exhibited 74.3 ± 3.5% of EtBr efflux inhibition, thus confirming its ability to inhibit NorA efflux pump. Indeed, we commonly consider as active NorA EPIs those compounds having an EtBr efflux inhibition ≥70%^17^^,^^18^.

### Time-kill curves

**Figure 3.**
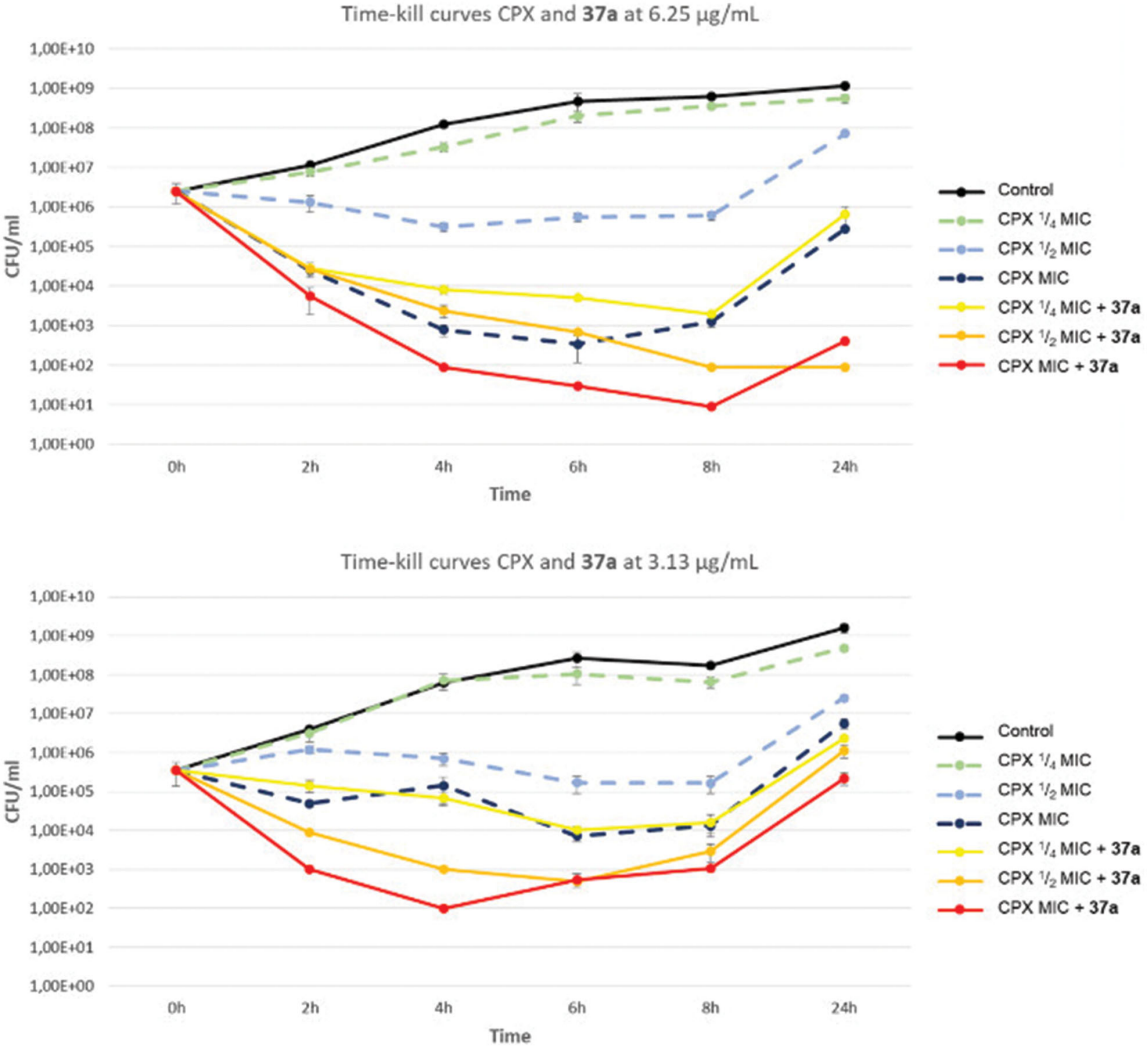
Time-kill curves of CPX and combination of compound **37a** with different concentrations of CPX against SA-1199B.

Subsequently, we decided to test the synergistic effect of compound **37a** (at 3.13 and 6.25 µg/mL) when combined with CPX in time-kill curve analysis against SA-1199B (Figure 3). Interestingly, at both concentrations, derivative **37a** exhibited a strong effect on the bactericidal activity of CPX, thereby proving its efficacy when combined with CPX over 24 h. Indeed, the combination of compound **37a** (at both the used concentrations) and CPX at ¼ MIC yielded the same bactericidal effect as CPX tested at its MIC. Moreover, the same concentrations of **37a** combined with CPX at its MIC showed a strongly significant and definitely higher bactericidal activity than that shown by CPX alone over the all 24 h. These findings highlight the high potential of the chlorothiophene derivative **37a** in potentiating CPX activity against resistant strains.

### Cytotoxicity assays

**Figure 4.**
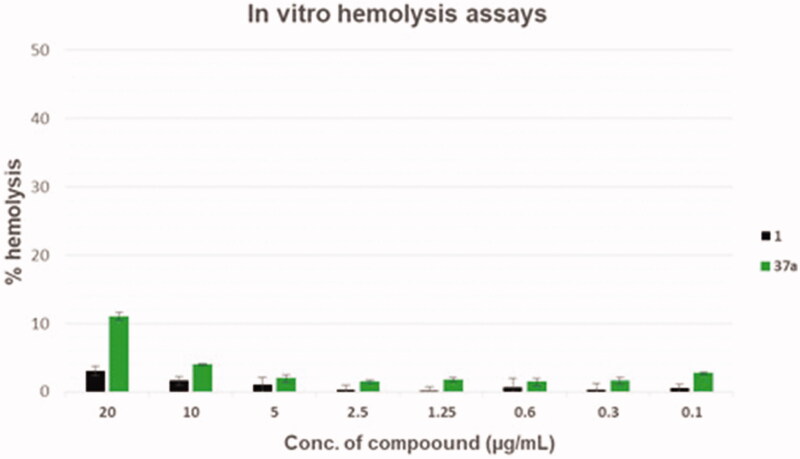
Haemolysis assays of compounds **37a** and starting hit **1**.

In order to evaluate the toxic effects of the derivative **37a**, haemolysis assays were performed at scalar compound concentrations in comparison with the starting hit **1** (Figure 4). Although derivative **37a** showed a slightly higher haemolytic effect than the starting hit **1**, when considering the activity-toxicity relationship, the chlorothiophene derivative **37a** exhibited an improved safety profile. In particular, **37a** at 20 µg/mL exhibited about 10% of haemolytic effect, a value 3-fold higher than the starting hit **1**, when tested at the same concentration. However, at concentrations ≤ 5 µg/mL (about 12-fold higher than that needed to reach synergism with CPX), compound **37a** reduced its haemolytic activity below 2%.

In addition, to further evaluate the toxic profile of compound **37a**, we determined its CC_50_ against human monocytic cells (THP-1); compound **1** was also tested for comparison (Table 1). To note, on THP-1 cells derivative **37a** exhibited a CC_50_ of 6.33 µg/mL, a concentration slightly lower than the CC_50_ of starting hit **1**. However, when compared with compound **2**, previously tested by us^19^, the toxicity of the chlorothiophene derivative **37a** resulted significantly increased. Therefore, it appeared evident that the replacement of the C-2 *p*OPr phenyl moiety (compound **1**) with a 5-chloro-2-thiophene portion (compound **37a**) led to a significant increase in the synergistic activity with CPX against SA-1199B while retaining the same toxic profile. Therefore, comparing both the concentrations needed to reduce by 4-fold CPX MIC and the respective CC_50_ values on THP-1 of **1** and **37a**, it was clear that **37a** exhibited a significant improvement in terms of “selectivity index” (16.2 for **37a** and 1.6 for **1**—Table 1). On the other hand, the presence of a –OMe substituent at the C-6 and an *O*-ethylpiperidine chain at C-4 of the quinoline core resulted essential to reduce the cytotoxicity against THP-1 cells as well as to confer a potent NorA EPI activity. Thus, thinking in terms of EPI activity, the introduction of the chlorothiophene moiety at the quinoline C-2 position is strongly recommended to boost the synergistic effect with CPX against SA-1199B; yet the increase in toxicity towards human cells should be considered. Therefore, these results could suggest further chemical modifications leading to new potent and safe quinoline-based NorA EPIs.

**Table 1.** Cytotoxicity assays against THP-1 cells of compounds **1**, **2** and **37a** and their calculated “selectivity index.”

Compound	CC_50_ (µg/mL)	SI^a^
**1**	10.0	1.6
**2**	>100^b^	>128
**37a**	6.33	16.2

^a^ SI, selectivity index calculated by the ratio between CC_50_ values and minimum EPI concentration able to reduce by 4-fold CPX MIC.

^b^ Data from Ref. 19.

### Membrane polarisation assays

Since efflux pumps need a proton gradient along the bacterial membrane, its modification/disruption can lead to an efflux inhibition. However, this effect cannot be related to a specific EPI activity; on the contrary, it relies on a non-specific interruption of the energy source (protons) that efflux pumps use to extrude their substrates by an antiport mechanism. A typical example of a compound inhibiting efflux pumps activity by this non-specific mechanism is represented by carbonyl cyanide *m*-chlorophenyl hydrazone (CCCP). Therefore, in order to know whether compound **37a** might counteract the NorA-mediated CPX efflux by disrupting proton motive force, we performed membrane polarisation assays against SA-1199B treated with **37a** at three different concentrations (1, 5, and 10 µg/mL); starting hit **1** (at the same concentrations) for comparison and CCCP at 1 µg/mL as positive control were included. Membrane polarisation was assessed by cytofluorimeter analysis using the fluorescent probe 3,3-diethyloxacarbocyanine iodide (DiOC_2_(3)) as its distribution is proton gradient-sensitive. DiOC_2_(3) in the presence of a bacterial membrane potential exhibits red fluorescence that shifts to green emission as the membrane potential is lost, thereby allowing calculation of the percentage of membrane polarisation by a red/green fluorescence ratio^41^.

Overall, the effect of the concentrations of **1** and **37a** on *S. aureus* membrane polarisation was dose-dependent. Indeed, both **1** and **37a** at 10 µg/mL extensively (>50%) depolarised *S. aureus* membrane. On the contrary, when **1** and **37a** were tested at 5 and 1 µg/mL, starting **1** retained a significant depolarising effect on the bacterial membrane while **37a** reduced its influence on the proton motive force. Indeed, at 5 and 1 µg/mL, compound **37a** was able to depolarise only about 20% of the bacterial membrane similarly to **2**, as previously reported by us^19^. Since **37a** retained a significant synergistic effect with CPX at a concentration as low as 0.39 µg/mL, we can observe that most of its activity is due to the NorA inhibition and not to a non-specific effect on the *S. aureus* membrane.

**Figure 5.**
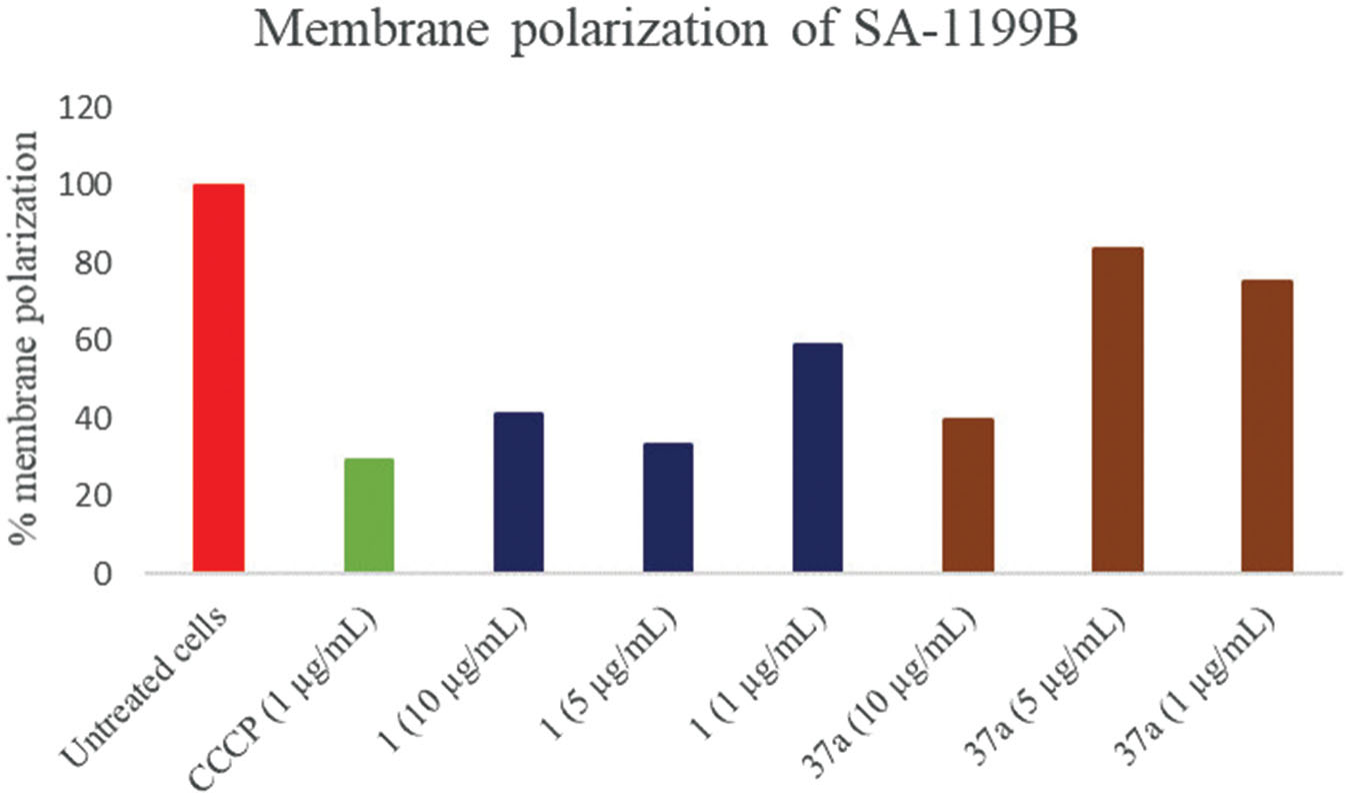
Membrane polarisation assays of compounds **1** and **37a** against SA-1199B at three different concentrations (1, 5 and 10 µg/mL) using the BacLight Bacterial Membrane Potential Kit. CCCP was used as positive control at 1 µg/mL (5 µM). % of membrane polarisation was calculated from the red/green fluorescence ratio by comparing bacterial cells in the presence of compounds with untreated cells.

Thus, the replacement of the *p*OPr-phenyl moiety of compound **1** with the chlorothiophene portion (compound **37a**), could reduce the depolarising effect on the bacterial membrane while increasing the synergistic effect with CPX; interestingly, this improvement in EPI activity can be related to a significant NorA inhibition thereby excluding an extensive disruption of the proton motive force (Figure 5).

### *In silico* ADME studies

We have previously demonstrated that our potent quinoline-based NorA EPI **2** exhibited a good metabolic stability in both *in silico* and *in vitro* experiments. However, demethylation of the C-6 methoxy group of **2** produced the most abundant metabolite after 2 h of incubation with mouse liver microsomes^19^.

**Figure 6.**
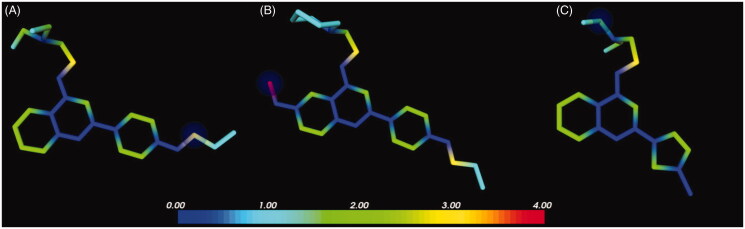
Predicted reactivity for compounds **1** (A), **2** (B) and **37a** (C). The atoms are colour-coded based on their predicted reactivity (red: high reactivity; blue: low reactivity). The blue sphere highlights the most probable site of metabolism.

Therefore, given the comparable EPI activity between derivatives **2** and **37a**, a prediction of the metabolic stability for the latter compound was planned by means of MetaSite software^33^, with the inclusion of the initial hit **1** and the potent derivative **2** for comparison. In agreement with the previous results^19^, the *in silico* protocol correctly predicted the methoxy moiety as the most reactive group as well as the most probable site of metabolism of quinoline **2** (Figure 6). By contrast, no highly reactive atoms were foreseen for derivatives **1** and **37a** (Figure 6). These results suggested that the chlorothiophene portion of compound **37a** represented a less reactive moiety than the *p*OPr chain present in derivatives **1** and **2**, underlining that the new potent EPI **37a** could couple the good biological activity of **2** with an enhanced metabolic stability. Indeed, the comparison between the NorA EPI activity and predicted metabolic stability of analogues **1**, **2** and **37a** showed that the use of the chlorothiophene moiety could lead to potent NorA quinoline-based EPIs missing the metabolic reactivity problems encountered with the introduction of the C-6 methoxy group as in derivative **2**.

In addition, it is noteworthy that **37a** obeyed the Lipinski’s rule of five, showing a lower molecular weight when compared to derivatives **1** and, in particular, **2** (361, 379 and 431 Da, respectively) and had computed physicochemical and pharmacokinetic descriptors in line with the recommended guidelines for orally dosed compounds (Table 2)^42^.

**Table 2.** Predicted physicochemical and ADME descriptors for derivative **37a**.

	Desidered value	**37a**
MW^a^	<500	361
Log*D*^b^	<5	3.62
Log*S*^c^	>1	1.41
HBA^d^	<5	2
HBD^e^	<10	1
tPSA (Å^2^)^f^	<180	26.6
Caco-2^g^	>–5	−4.48
HIA^h^	+	+
2C9 p*K_i_*^i^	<6	5.02
Stability^j^	Stable	Stable

^a^ Molecular weight.

^b^ Logarithm of the octanol/water partition coefficient at pH = 7.4.

^c^ Intrinsic aqueous solubility: log*S* ≥ 1 corresponds to intrinsic aqueous solubility greater than 10 µM.

^d^ Number of hydrogen bond acceptors.

^e^ Number of hydrogen bond donors.

^f^ Topological polar surface area.

^g^ Apparent permeability across monolayers of the Caco-2 line of human epithelial colorectal adenocarcinoma cells.

^h^ Human intestinal adsorption: classification of “+” for compounds which are ≥30% adsorbed and “−” for compounds which are <30% adsorbed.

^i^ p*K*_i_ values for CYP2C9 affinity. The defined threshold is to avoid drug-drug interactions due to inhibition of CYP2C9.

^j^ Human liver microsomal stability. All property values were calculated using Optibrium in SeeSAR^34^^,^^36^.

## Conclusions

In order to identify new potent EPIs to counteract the rapid insurgence of bacterial resistance towards common antibiotics, we made a further effort to build a robust SAR delineation around the quinoline-based NorA inhibitors. Herein, we have described the design, synthesis and biological evaluation of new 2-arylquinoline derivatives. In particular, a new chlorothiophene analogue (**37a**) endowed with high synergistic effect with CPX against SA-1199B, was identified. To balance the lack of biophysical experiments proving compound binding to NorA pump and in an attempt to exclude potential non-specific effects, we carried out further experiments supporting that a NorA inhibition could produce the observed synergistic activity of **37a**. Indeed, the demonstration that our compound did inhibit EtBr efflux in a phenotypic assay and did not extensively depolarise *S. aureus* membrane strongly underpinned that **37a** can specifically inhibit NorA. In addition, time-kill curves of this EPI combined with CPX displayed the high potential of an EPI in boosting CPX bactericidal effect.

Data collected on 2-arylquinoline derivatives will be useful to definitely obtain a set of compounds having potent NorA EPI activity, poor non-specific effects on bacterial membrane and an acceptable cytotoxic profile in order to justify the use of these EPIs in animal models of infections.
